# MiRNA Expression Profile in the Airways Is Altered during Pulmonary Exacerbation in Children with Cystic Fibrosis—A Preliminary Report

**DOI:** 10.3390/jcm9061887

**Published:** 2020-06-16

**Authors:** Zuzanna Stachowiak, Irena Wojsyk-Banaszak, Katarzyna Jończyk-Potoczna, Beata Narożna, Wojciech Langwiński, Zdzisława Kycler, Paulina Sobkowiak, Anna Bręborowicz, Aleksandra Szczepankiewicz

**Affiliations:** 1 Molecular and Cell Biology Unit, Department of Paediatric Pulmonology, Allergy and Clinical Immunology, Poznan University of Medical Sciences, 60-572 Poznań, Poland; zuzastachowiak9@gmail.com (Z.S.); b.narozna@gmail.com (B.N.); wlangwinski654@gmail.com (W.L.); 2 Department of Paediatric Pulmonology, Allergy and Clinical Immunology, Poznan University of Medical Sciences, 60-572 Poznań, Poland; iwojsyk@ump.edu.pl (I.W.-B.); kyclerzdzislawa@interia.pl (Z.K.); paulina-25@tlen.pl (P.S.); abreborowicz@wp.pl (A.B.); 3 Department of Paediatric Radiology, Poznan University of Medical Sciences, 60-572 Poznań, Poland; potocznak@op.pl

**Keywords:** miRNA expression, exhaled breath condensate, sputum, severity, pulmonary exacerbation

## Abstract

MicroRNAs are small non-coding RNAs that regulate immune response and inflammation. We assumed that miRNAs may be involved in the immune response during cystic fibrosis pulmonary exacerbations (CFPE) and that altered expression profile in the airways and blood may underlie clinical outcomes in CF pediatric patients. Methods: We included 30 pediatric patients diagnosed with cystic fibrosis. The biologic material (blood, sputum, exhaled breath condensate) was collected during pulmonary exacerbation and in stable condition. The miRNA expression profile from blood and sputum (*n* = 6) was done using the next-generation sequencing. For validation, selected four miRNAs were analyzed by qPCR in exosomes from sputum supernatant and exhaled breath condensate (*n* = 24). NGS analysis was done in Base Space, correlations of gene expression with clinical data were done in Statistica. Results: The miRNA profiling showed that four miRNAs (miR-223, miR-451a, miR-27b-3p, miR-486-5p) were significantly altered during pulmonary exacerbation in CF patients in sputum but did not differ significantly in blood. MiRNA differently expressed in exhaled breath condensate (EBC) and sputum showed correlation with clinical parameters in CFPE. Conclusion: MiRNA expression profile changes in the airways during pulmonary exacerbation in CF pediatric patients. We suggest that miRNA alterations during CFPE are restricted to the airways and strongly correlate with clinical outcome.

## 1. Introduction

Cystic fibrosis (CF) is a common autosomal recessive disorder caused by mutations in the cystic fibrosis transmembrane conductance regulator (CFTR) gene. It is a multiorgan disease that affects the pancreas, gastrointestinal and reproductive tracts and lungs [1]. Common pulmonary symptoms include deposition of thick mucus that leads to severe airflow obstruction, chronic inflammation and chronic airway infection [2]. Lung disease is characterized by intermittent episodes of acute worsening of respiratory symptoms, pulmonary exacerbations (CFPE) [3] that cause lung function decline and contribute to disease progression, poor quality of life and shortened life expectancy in CF patients.

Retention of thick mucus in the airways facilitates frequent pulmonary infections (caused by such pathogens as *P. aeruginosa*, *S. aureus*, *H. influenzae*) [4]. However, pulmonary exacerbations do not usually result from increased bacterial density within the airways [5,6], and more severe symptoms are caused by chronic enhanced airway inflammation that may even precede infection. Chronic inflammation in the airways leads to airway epithelium damage and activates repair processes contributing to remodeling and subepithelial fibrosis that further impair lung function. Current CFPEs are the leading cause of morbidity and mortality [7,8], indicating the need for biomarker discovery.

Published data looking for CFPE biomarkers either in blood or the airways suggest many immunologically active molecules, such as TNFα, IL-8, myeloperoxidase (MPO), calprotectin, C reactive protein (CRP), correlated with clinical deterioration [9,10]. However, none of them were specific for CF pulmonary exacerbation. Moreover, peripheral biomarkers may not truly reflect the local inflammatory response in the lungs [11]. Available data suggest that sputum is a reliable source of inflammatory biomarkers, including circulating miRNA [12,13]. Less is known about the markers in exhaled breath condensate (EBC) [14].

MicroRNA are small (21–23 bp), non-coding RNAs that modulate the expression of various proteins via post-transcriptional inhibition of gene expression. They are well-known regulators of immune responses and chronic inflammation in respiratory diseases, including asthma, COPD or idiopathic pulmonary fibrosis (as reviewed by [15,16]). Moreover, they are expressed not only inside the cells but are also stably present in extracellular space i.e., body fluids such as plasma, sputum, bronchoalveolar lavage fluid (BALF) and EBC. Previous studies of miRNA in the course of CF reported a few miRNAs associated with CFTR activity (either decreased expression e.g., miR-509-3p, miR-145, miR-223 or increased expression, e.g., miR-138) [17] as well as a pulmonary exacerbation. Krause et al. studied the latter and found that the expression of the Mirc1/Mir17–92 cluster correlated with pulmonary exacerbation, but only in sputum samples and not in plasma [18]. Altered miRNA expression also affects innate immune responses in the CF lung. Previous reports showed that mir-126 was significantly downregulated in CF bronchial brushings and thus influenced the expression of Target of Myb1 (TOM1) membrane trafficking protein [19,20]. However, previous studies on the role of miRNA in the pediatric CF population are limited. The recent analysis focused on the miRNA expression profile and gender differences in CF children [21], but the studies investigating miRNA expression profile in CF pulmonary exacerbation are lacking.

Taking into account that miRNAs are regulators of immune inflammatory responses and that enhanced chronic inflammation plays a significant role in pulmonary exacerbations in cystic fibrosis, we aimed to investigate if miRNA expression differs between exacerbation and stable period of disease and if altered expression profile in the airways may underlie clinical outcome in CF pediatric patients.

## 2. Materials and Methods

### 2.1. Study Design

The prospective cohort of 30 pediatric patients, girls and boys aged 6–18 years diagnosed with cystic fibrosis and admitted to the Department of Pulmonology, Pediatric Allergy and Immunology, was enrolled in the project. Patients were either experiencing a pulmonary exacerbation or in a stable stage of disease (control visit). The Bioethics Committee approved the study (no. 386/17). All pediatric participants and their parents gave written consent.

### 2.2. Clinical Analysis

CF was diagnosed based on typical clinical presentation, two positive sweat chloride tests and the presence of two pathogenic mutations of CFTR. Pulmonary exacerbation was defined according to the EuroCareCF working group [22,23]. In all patients, we assessed: blood morphology, inflammatory proteins (CRP), microbiologic sputum culture, chest imaging (Brasfield score), spirometry and disease severity (with Shwachman–Kulczycki score). The data were collected, as described previously [24].

Sputum samples were collected during spontaneous expectoration. Samples were prepared by the addition of Sputolysin (Merck, Kenilworth, NJ, USA) and centrifuged to separate the cell pellet from the supernatant. For further analysis, sputum supernatant was used. We used the Turbo DECCS device (Medivac) to collect EBC according to ERS recommendation [25]. From all patients, the same amount (3 mL) of EBC was collected. Whole blood samples were drawn to tubes with EDTA anticoagulant, and after the immediate addition of Lysis Buffer (Macherey–Nagel, Düren, Germany), samples were frozen at −80 °C for further analysis.

### 2.3. Molecular Analysis

MiRNA samples from blood and sputum were extracted using NucleoSpin RNA blood kit (Macherey–Nagel). Exosomes from sputum supernatant and exhaled breath samples were precipitated using a miRCURY exosome cell/urine/CSF Isolation Kit (Qiagen, Hilden, Germany). MicroRNA was extracted using RNeasy mini kit (Qiagen) and RNeasy MinElute spin columns according to protocol to obtain miRNA fraction. The quantity of miRNA in the samples was measured using microRNA assay kit (Thermo Fisher Scientific, Waltham, MA, USA). The miRNA expression profile from the whole blood and sputum samples of 6 CF patients during exacerbation and stable period was performed using next-generation sequencing. Libraries were generated from 50 ng of miRNA sample using TruSeq small RNA library preparation kit (Illumina, San Diego, CA, USA) following the manufacturer’s instructions. Libraries were validated and quantified using high sensitivity DNA screen tape (Agilent, Santa Clara, CA, USA) on Tape Station 2200 (Agilent) and run on MiniSeq sequencer (Illumina) with 50-nt single-end reads. Differential miRNA expression analysis was done in Base Space software. The miRNA genes that showed the most significant differences between CF exacerbation and stable period in miRNA expression profiling from sputum were analyzed using qPCR in the validation CF cohort (*n* = 24 samples) in sputum supernatants and exhaled breath condensate. For reverse transcription, we used MystiCq microRNA cDNA Synthesis Mix (Sigma-Aldrich, St. Louis, MO, USA). Quantitative PCR was done using MystiCq microRNA SYBR Green qPCR ReadyMix (Sigma Aldrich) and microRNA assays for miR-486-5p, miR-223, miR-451a, miR-27b. Expression analysis was conducted on the 7900HT fast-real time PCR system (Applied Biosystems, Foster City, CA, USA). Differential expression results from qPCR were compared using Data Assist software (Thermo Fisher Scientific) with a relative quantification method based on the global normalization algorithm.

### 2.4. Statistical Analysis

The data distribution was analyzed using the Shapiro–Wilk test. Normally distributed data were analyzed using parametric tests and data that deviated from normal distribution were analyzed using nonparametric tests. The correlation of miRNA expression and interval variables (lung function parameters, Shwachman–Kulczycki score, Brasfield score, neutrophil counts, CRP level) compared between exacerbation and stable period was done using the Spearman correlation test. The comparison of miRNA expression and nominal variables (presence of bacterial or fungal infection, presence of exacerbation) was performed using analysis of variance (one-way or two-way ANOVA) in Statistica v.12 (Statsoft, Cracow, Poland).

## 3. Results

### 3.1. A Clinical Description of the Analyzed Group

Clinical characteristics of the patients based on biologic material (exhaled breath condensate, sputum supernatant) are given in Table 1 and Table 2. The exacerbation group was similar in age and gender to the patients during a stable period. Chronic *Pseudomonas aeruginosa* colonization was rarely observed in our population (≤25%) and not significantly different between exacerbation vs. stable groups. The lung function between groups was not significantly different, but the groups differed in clinical outcomes such as disease severity (SK score, Brasfield score). The treatment was uniform for the CF patients, depending on clinical symptoms. Patients chronically infected with *Pseudomonas aeruginosa* were treated with inhaled antibiotics, mostly colistimethate sodium, and two patients received inhaled tobramycin. All the patients received dornase alfa, all patients with pancreatic insufficiency received pancreatic enzymes and none were treated with CFTR modulators.

### 3.2. Comparison of miRNA Expression Profile between Exacerbation and Stable Period

**Figure 1:**
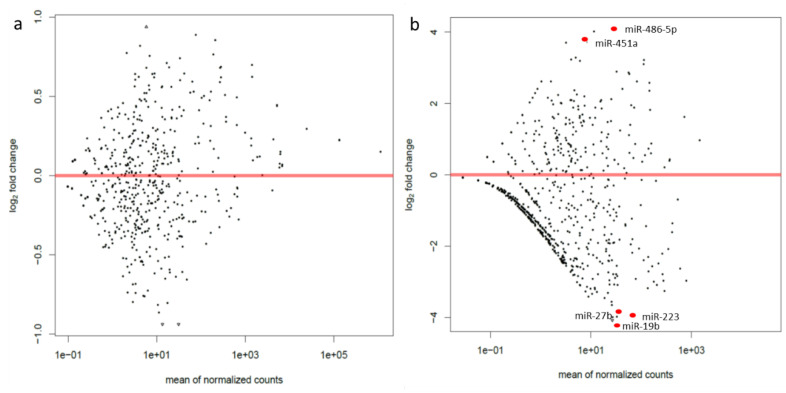
MA-plot between exacerbation and stable period of the gene expression counts (**a**) for the whole blood and (**b**) for sputum. Significantly altered miRNAs are marked as red dots (DeSeq).

Next-generation sequencing of the whole blood miRNA profile showed that out of 186 miRNAs expressed in blood, CF patients did not differ significantly between exacerbation and the stable period in (Figure 1a). Comparative analysis of the miRNA expression profile in sputum showed significant differences in the expression of 9 miRNAs (including mature miRNAs: miR-223-3p, miR-451a, miR-27b-3p and miR-486–5p and their isomiRs: 2 isomiRs of miR-486–5p, one of miR-451a and one of miR-223-3p.) between exacerbation and stable period out of 158 expressed in sputum (Figure 1b). Three of them, miR-27b, miR-223 and miR-19b, showed a decrease, whereas the other two (miR-486-5p, miR-451a) showed increased expression during exacerbation.

Four mature miRNAs differentially expressed in sputum (miR-223-3p, miR-451a, miR-27b-3p and miR-486–5p) were selected for qPCR analysis in the sputum supernatant from 24 patients and exhaled breath condensate samples from 24 patients. One miRNA, miR-19b, showed very low expression in the sequencing experiment, so we excluded it from qPCR validation. During exacerbation, we observed the highest increase in expression for miR-486–5p in both EBC and sputum supernatant samples, but the difference compared to a stable period was not significant. For miR-223-3p, we found that decreased expression during exacerbation in the sequencing experiment was not confirmed in the validation cohort.

Expression of exosomal miR-223-3p was higher in CF patients during exacerbation in EBC and sputum samples, but the difference was not significant (*p* > 0.05). In EBC during exacerbation miR-451a was more abundant than in stable period, but the difference was not statistically significant (*p* = 0.41). In sputum supernatant, miR-451a was decreased during exacerbation than stable period, yet the difference did not reach significance. The only miRNA that showed decreased expression in both EBC and sputum samples during exacerbation was miR-27b, but this decrease was not significant. The results are shown in Table 3.

### 3.3. Correlation of miRNA Expression with Clinical Parameters

#### 3.3.1. MiR-223-3p

**Figure 2:**
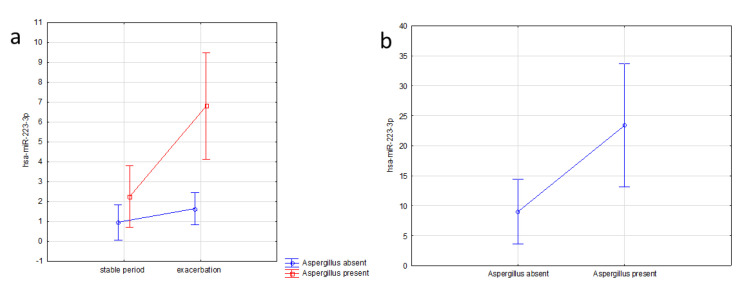
The association of miR-223-3p expression and clinical parameters in CF patients (**a**) in EBC in relation to *Aspergillus* infection and exacerbation (F = 5.953, *p* = 0.024, 2-way analysis of variance); (**b**) in sputum supernatant in relation to *Aspergillus* infection (F = 6.714, *p* = 0.017, one-way analysis of variance).

The higher expression of this miRNA in EBC correlated with *Aspergillus* infection in the airways during pulmonary exacerbation in CF patients (*p* = 0.02). Similar to EBC, we observed higher levels of this miRNA in sputum supernatants in children infected with *Aspergillus* (*p* = 0.08) (Figure 2), but not with symptoms severity (Shwachman–Kulczycki score, Brasfield score) or lung function.

#### 3.3.2. MiR-451a

**Figure 3:**
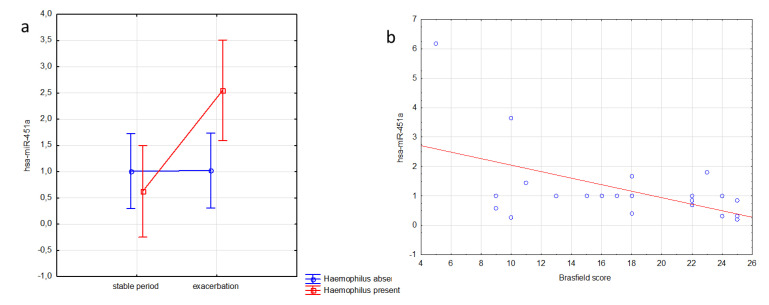
Association of miR-451a expression in EBC and clinical parameters in CF patients, (**a**) in relation to *Hemophilus* infection and exacerbation (F = 5.760; *p* = 0.024, 2-way analysis of variance); (**b**) correlation with the Brasfield score (r = −0.526; *p* = 0.001, Spearman correlation).

We observed that CF patients infected with *H. influenzae* had significantly higher miR-451a EBC expression during exacerbation (*p* = 0.02). In contrast, the level of expression in uninfected CF patients was similar and independent of exacerbation. We also observed a significant inverse correlation between miR-451a EBC expression and the Brasfield score (r^2^ = −0.53, *p* = 0.01) (Figure 3), but the other clinical parameters did not correlate with the expression of this miRNA. We found no significant differences in miR-451a expression with clinical parameters in sputum.

#### 3.3.3. MiR-27b-3p

The expression of miR-27b in EBC positively correlated with lung function parameters: FEV1 (r^2^ = 0.669, *p* = 0.077) and FVC (r^2^ = 0.637, *p* = 0.006) during pulmonary exacerbation.

**Figure 4:**
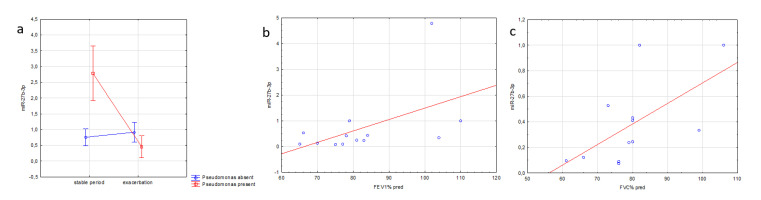
Association of miR-27b expression and clinical parameters in CF patients, (**a**) in sputum correlation with *Pseudomonas* infection during exacerbation (F = 25.267, *p* < 0.001, 2-way analysis of variance); (**b**) in EBC correlation with FEV1 (r = 0.510; *p* = 0.077) and (**c**) FVC (r = 0611; *p* = 0.034) during exacerbation (Spearman correlation).

Current infection with *Pseudomonas aeruginosa* was associated with decreased expression of miR-27b-3p in sputum supernatant during exacerbation than the patients in a stable period (*p* < 0.0001). It remained unaltered in patients without *Pseudomonas* infection (Figure 4).

#### 3.3.4. MiR-486-5p

**Figure 5:**
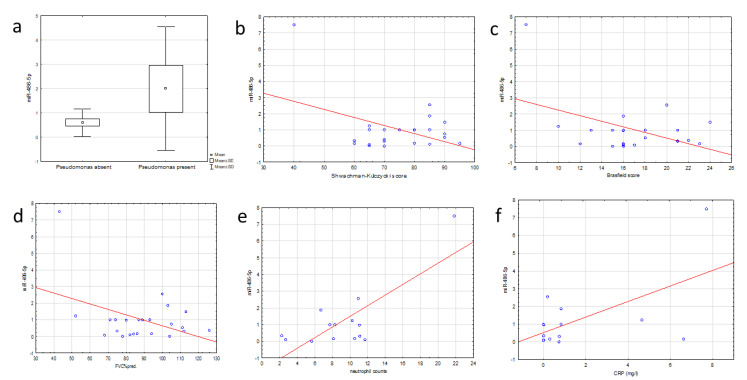
Association of miR-486-5p expression in sputum and clinical parameters in CF patients (**a**) in relation to *Pseudomonas* infection (*p* = 0.044, 1-way analysis of variance); (**b**) correlation with Shwachman–Kulczycki score (r = −0.42, *p* = 0.048), (**c**) correlation with Brasfield score (r = −0.45, *p* = 0.031); (**d**) Spearman correlation with FVC (r = −0.451, *p* = 0.045); correlation with peripheral markers of inflammation: (**e**) neutrophil counts (r = 0.772; *p* = 0.001) and (**f**) CRP levels (r = 0.592; *p* = 0.025) during exacerbation (Spearman correlation).

The expression of this miRNA in EBC did not correlate significantly with analyzed clinical parameters in CF patients. In sputum, miR-486-5p expression was higher in CF patients colonized with *Pseudomonas aeruginosa* (*p* = 0.044). Further analysis showed an inverse correlation between this miRNA and Shwachman–Kulczycki score (r^2^ = −0.419, *p* = 0.047) as well as the Brasfield score (r^2^ = −0.459, *p* = 0.031) and lung function (FVC) (r^2^ = −0.421, *p* = 0.046). On the other hand, results for two parameters of peripheral inflammation, CRP and neutrophilia, positively correlated with miR-486-5p expression in CF patients during PE for CRP (r^2^ = 0.592, *p* = 0.027) and neutrophilia (r^2^ = 0.772, *p* = 0.001) (Figure 5).

## 4. Discussion

The main finding of this study is the altered miRNA expression profile in the airways, but not in blood, during pulmonary exacerbation. We also found that altered miRNAs (miR-223-3p, miR-451a, miR-27b, miR486-5p) correlate with clinical outcomes (bacterial infections, the severity of symptoms, lung function, peripheral inflammation) in sputum and exhaled breath condensate of CF patients.

Previous studies showed that miR-223 is a negative regulator of neutrophil activation and chemotaxis in experimental models of inflammatory diseases. This miRNA mediates intracellular neutrophil action as well as extracellular inflammation responses in the lungs [26,27]. In CF, neutrophilic inflammation in the lung correlated with more severe lung disease [28,29]. Previous data indicated that *Aspergillus* colonization may be associated with reduced lung function and that *Aspergillus* infection increased neutrophil count and elevated the levels of inflammatory cytokines (e.g., IL-8) in BALF [30,31]. In our study, we found increased expression of miR-223 during exacerbation with concurrent *Aspergillus* infection. Thus, miR-223-3p upregulation may be a response to infection and enhanced airway inflammation induced by *Aspergillus*.

Regarding miR-451a, a previous study showed increased expression of this miRNA in BALF from healthy subjects in the lower lung lobes compared to the upper lobes [32]. In silico analysis indicated several targets for miR-451a, e.g., macrophage migration inhibitory factor (MIF) and matrix metalloproteinase-2 (MMP-2), that are innate immunity mediators [33]. MIF is a proinflammatory molecule that enhances Gram-negative inflammatory responses, and its high level accelerates end-organ injury in CF [33]. In our study, we found the highest miR-451a expression in EBC during exacerbation in patients infected with *Hemophilus influenzae*. Infection with these Gram-negative bacteria in cystic fibrosis often co-occurs with PE [34]. An inverse correlation of miR-451a expression with the Brasfield score suggests that increased expression in the upper airways (EBC) may be a marker of disease progression assessed by radiological changes. Interestingly, the expression of miR-451a in CF exacerbation slightly decreased in sputum and did not correlate with *Hemophilus* infection. This observation indicates that miRNA alterations are site-specific within the respiratory tract and differ between body fluids from the upper and lower airways.

The expression of miR-27b-3p decreased during pulmonary exacerbation in both EBC and sputum supernatant, however, this change was not significant. Previous studies indicated this miRNA silences the immune response by reducing cytokine secretion, thus inhibiting local macrophage recruitment. Its expression increased during acute infection, e.g., sepsis preventing the spreading of inflammation [35]. This action was confirmed in an animal model of *Mycobacterium tuberculosis* infection [36]. Infected mice overexpressed in the airways miR-27b that suppressed the production of pro-inflammatory factors and excessive inflammation. Similarly, we observed that *Pseudomonas aeruginosa* infection correlated with overexpression of miR-27b in sputum in a stable period. Thus, its increase seems to suppress the exaggerated inflammatory response. Its significant decrease during exacerbation correlated with enhanced inflammation induced by *Pseudomonas* infection. Increased expression of miR-27b in EBC also associated with better lung function parameters, e.g., FEV_1_ or FVC, suggesting that airway inflammation suppressed by miR-27b overexpression may translate into improved lung function in CF.

Previous data regarding mir-486–5p showed its increased plasma expression in CF patients than healthy controls [37]. We confirmed its expression in whole blood and also in sputum and EBC. A recent study showed that mice with induced acute lung injury presented higher miR-486-5p expression, whereas its inhibition with antagomir blocked inflammatory response and apoptosis, thus reducing lung injury [38]. Our results showed an increased expression of exosomal miR-486-5p in the sputum of CF patients infected with *P. aeruginosa*. Its expression inversely correlated with symptoms severity (Shwachman–Kulczycki and Brasfield scores) and lung function indicating a worse course of lung disease. Therefore, the increased local expression of this microRNA may be a marker of worse prognosis in CF patients. However, further studies are needed to confirm this assumption.

One of the main limitations of the study is small sample size of our group and the fact that patients from the exacerbation group were different from patients from the stable group.

Another limitation may be high variability in clinical and miRNA expression data comparisons in the correlation analysis and to address this issue, we used nonparametric tests to minimize the influence of outliers on the results interpretation.

## 5. Conclusions

In conclusion, we found the altered miRNA expression profile in the airways of CF patients. These changes correlate with the clinical outcomes and suggest the involvement of miRNA regulation in pulmonary exacerbation. Our study also indicates the potential of non-invasively collected material from the airways (exhaled breath condensate) that may be a source of extracellular biomarkers of CF pulmonary exacerbation in children.

## Figures and Tables

**Table 1:** Characteristics of the Cystic fibrosis (CF) patients (exhaled breath condensate (EBC) samples).

Mean (±SD) or Number (%)			
Variable	Stable	Exacerbation	*p* Value
Number of subjects	12	12	-
Female	6 (50%)	7 (58%)	1.000
Age (years)	12.4 (±3.5)	13.0 (±3.4)	0.706
F508 del homozygous	4 (33%)	6 (50%)	0.680
F508del heterozygous	5 (42%)	2 (17%)	0.371
BMI (kg/m^2^)	17.8 (±2.0)	17.1 (±2.3)	0.421
Diabetes (including under observation)	3 (25%)	5 (42%)	0.667
Pancreatic insufficiency	10 (83%)	11 (92%)	1.000
*P. aerugionsa* colonization	2 (17%)	4 (33%)	0.365
*P. aeruginosa* current infection	0 (0%)	3 (25%)	0.217
*S. aureus* colonization	9 (75%)	11 (92%)	0.590
FEV_1_% pred.	98.3 (±19.8)	82.8 (±14.9) 84.8 (±12.5) *	0.041 0.058
FVC% pred.	96.6 (±14.5)	82.9 (±16.4) 83.2 (±13.3) *	0.041 0.027
Shwachman–Kulczycki score (median)	90.0 (65.0–95.0)	65.0 (40.0–85.0)	0.001
Brasfield score (median)	23.0 (11.0–25.0)	13.0 (5.0–22.0)	0.009

* spirometry baseline result. BMI–body mass index; FEV1–forced expiratory volume in the first second, FVC–forced vital capacity.

**Table 2:** Characteristics of the CF patients (sputum samples).

Mean (±SD) or Number (%)			
Variable	Stable	Exacerbation	*p* Value
Number of subjects	11	13	-
Female	5 (45%)	6 (43%)	1.000
Age (years)	11.8 (±3.1)	13.7 (±3.8)	0.200
F508 del homozygous	5 (45%)	10 (71%)	0.241
F508del heterozygous	3 (27%)	3 (21%)	1.000
BMI (kg/m^2^)	18.5 (±3.1)	18.7 (±2.4)	0.885
Diabetes (including under observation)	4 (36%)	7 (50%)	0.689
Pancreatic insufficiency	9 (81%)	12 (92%)	0.615
*P. aeruginosa* colonization	2 (18%)	0 (0%)	0.113
*P. aeruginosa* current infection	2 (18%)	6 (43%)	0.234
*S. aureus* colonization	8 (73%)	13 (93%)	0.288
FEV_1_% pred.	96.0 (±20.7)	79.6 (±24.6) 84.2 (±22.2) *	0.095 0.194
FVC% pred.	94.8 (±15.1)	83.2 (±19.4) 86.9 (±18.1) *	0.121 0.263
Shwachman–Kulczycki score (median)	90.0 (60.0–95.0)	70.0 (40.0–85.0)	0.009
Brasfield score	20.2 (±3.3)	15.4 (±3.9)	0.006

* spirometry baseline result. BMI–body mass index; FEV1–forced expiratory volume in the first second, FVC–forced vital capacity.

**Table 3:** **MicroRNA** (MiRNA) levels in patients during pulmonary exacerbation in reference to stable period.

miRNA	Expression (EBC)	*p* Value	Expression (Sputum)	*p* Value
miR-223-3p	**↑**	0.177	**↑**	0.411
miR-451a	**↑**	0.342	**↓**	0.406
miR-27b-3p	**↓**	0.160	**↓**	0.312
miR-486-5p	**↑**	0.656	**↑**	0.341
